# Anethole Pretreatment Modulates Cerebral Ischemia/Reperfusion: The Role of JNK, p38, MMP-2 and MMP-9 Pathways

**DOI:** 10.3390/ph16030442

**Published:** 2023-03-15

**Authors:** Nancy S. Younis, Maged E. Mohamed

**Affiliations:** 1 Department of Pharmaceutical Sciences, College of Clinical Pharmacy, King Faisal University, Al-Ahsa 31982, Saudi Arabia; 2 Zagazig University Hospitals, Zagazig University, Zagazig 44519, Egypt; 3 Department of Pharmacognosy, College of Pharmacy, Zagazig University, Zagazig 44519, Egypt

**Keywords:** anethole, cerebral ischemia/reperfusion, blood–brain barrier integrity, infarct volume, JNK/p38 pathway

## Abstract

Anethole (AN) is one of the major constituents of several plant oils, demonstrating plentiful pharmacological actions. Ischemic stroke is the main cause of morbidity and death worldwide, particularly since ischemic stroke therapeutic choices are inadequate and limited; thus, the development of new therapeutic options is indispensable. This study was planned to explore the preventive actions of AN in ameliorating cerebral ischemia/reperfusion-induced brain damage and BBB permeability leakage, as well as to explore anethole’s potential mechanisms of action. The proposed mechanisms included modulating JNK and p38 as well as MMP-2 and MMP-9 pathways. Sprague–Dawley male rats were randomly assigned into four groups: sham, middle cerebral artery occlusion (MCAO), AN125 + MCAO, and AN250 + MCAO. Animals in the third and fourth groups were pretreated with AN 125 or 250 mg/kg orally, respectively, for two weeks before performing middle cerebral artery occlusion (MCAO)-induced cerebral ischemic/reperfusion surgery. Animals that experienced cerebral ischemia/reperfusion exhibited amplified infarct volume, Evans blue intensity, brain water content, Fluoro-Jade B-positive cells, severe neurological deficits, and numerous histopathological alterations. MCAO animals exhibited elevated MMP-9 and MMP-2 gene expressions, enzyme activities, augmented JNK, and p38 phosphorylation. On the other hand, pretreatment with AN diminished the infarct volume, Evans blue dye intensity, brain water content, and Fluoro-Jade B-positive cells, improved the neurological score and enhanced histopathological examination. AN effectively lowered MMP-9 and MMP-2 gene expression and enzyme activities and diminished phosphorylated JNK, p38. AN decreased MDA content, amplified GSH/GSSG ratio, SOD, and CAT, decreased the serum and brain tissue homogenate inflammatory cytokines (TNF-α, IL-6, IL-1β), NF-κB, and deterred the apoptotic status. This study revealed the neuroprotective ability of AN against cerebral ischemia/reperfusion in rats. AN boosted blood–brain barrier integrity via modulating MMPs and diminished oxidative stress, inflammation, and apoptosis through the JNK/p38 pathway.

## 1. Introduction

Anethole, 1-methoxy-4-(1-propenyl) benzene, (AN) is one of the major constituents of several plant essential oils, including anise, fennel, and camphor oils [1]. AN offered numerous pharmacological actions including anti-hypernociceptive (analgesic) and anti-inflammatory activity [2,3], immunomodulatory [4], antioxidant [5], gastroprotective [5], hypotension and bradycardia [6], antithrombotic and vasorelaxant [7] abilities. AN relieved cellular and vascular inflammation actions, thus controlling both nonimmune inflammatory conditions [8] as well as several immune–inflammatory conditions such as inflammatory pain [2] and LPS-induced periodontitis [9]. Furthermore, AN exerted anti-metastatic activity in human breast cells (MCF-7, MDA-MB-231) [10], cervical carcinoma [11], and prostate cancer cells [12], and enhanced the efficiency of some chemotherapeutic drugs such as cyclophosphamide [13], tamoxifen [14], as well as decreasing chemotherapy side effects [15]. AN, in a dose of 10 μM, significantly reduced neuronal cell death, which was induced by oxygen-glucose deprivation/reoxygenation through the antioxidant and anti-excitotoxic effects as well as mitochondrial protection [16]. Furthermore, AN exhibited beneficial capabilities in neurodegenerative diseases such as Alzheimer’s and Parkinson’s. AN showed acetylcholinesterase-inhibiting activity, and therefore the compound played an important role in the deterrence of cognitive impairment associated with cholinergic insufficiency [17]. AN interfered with several essential signaling pathways, including the nuclear factor k-light-chain enhancer of activated B cells (NFκB) [18], MAPK (mitogen-activated protein kinase) [18], STAT (signal transducer and activator of transcription) [19] and AP-1 (activator protein-1), as well as cytokines signaling TNF-α [18], interferon (IFN)-γ [20], or matrix-metalloproteinase (MMPs) activities [21].

Ischemic stroke is the main cause of morbidity and death worldwide, especially since the ischemic stroke management therapeutic choices are inadequate and limited; thus, the development of new therapeutic options is indispensable [22]. Cerebral ischemia/reperfusion (C I/R)-induced damage occurs in two steps process. First, neurons damage and/or die due to ischemic stroke, followed by reestablishing blood flow to the ischemic zones, aggravating brain damage and further deteriorating the condition [23]. During C I/R, the blood–brain barrier (BBB) permeability amplifies, which promotes cerebral edema [24]. As a result of brain volume expanding, the intracranial pressure elevates [25], and the inflammatory cells and inflammatory factors permit into intensifying inflammatory and apoptosis reactions, which cause further deterioration of the disease [26]. Thus, BBB destruction plays a crucial part in the pathophysiology of cerebral ischemia/reperfusion. Matrix-metalloproteinase-2 and -9 (MMP-2 and MMP-9) are upregulated during ischemic stroke [27,28]. These elevated MMPs are associated with BBB permeability disruption with subsequent brain edema development with cerebral ischemia/reperfusion [29].

Mitogen-activated protein kinase (MAPK) signaling mediates countless imperative cellular processes, including cell growth and survival [30,31]. The three main MAPK signaling pathways in mammalian species are c-Jun N-terminal kinase (JNK), p38 MAPK, and extracellular signal-regulated protein kinase (ERK) [32]. The stress-activated protein kinases JNK and p38 are motivated by cytokines, death receptors, mitogens, and cellular stress, including oxidative stress, heat shock, ultraviolet irradiation, and others [33]. The JNK and p38 pathways play crucial roles in regulating apoptosis, inflammation, and cell-cycle arrest [34]. 

This study plans to explore the preventive actions of AN in ameliorating focal cerebral ischemia/reperfusion-induced brain damage and BBB permeability leakage in rats and to investigate AN’s potential mechanisms of action. The proposed mechanisms included modulating JNK and p38 as well as MMP-2 and MMP-9 with subsequent effects on oxidative stress, inflammation, and apoptosis.

## 2. Results

### 2.1. AN Alleviated Brain Injury Caused by Middle Cerebral Artery Occlusion (MCAO)-Induced C I/R

**Figure 1:**
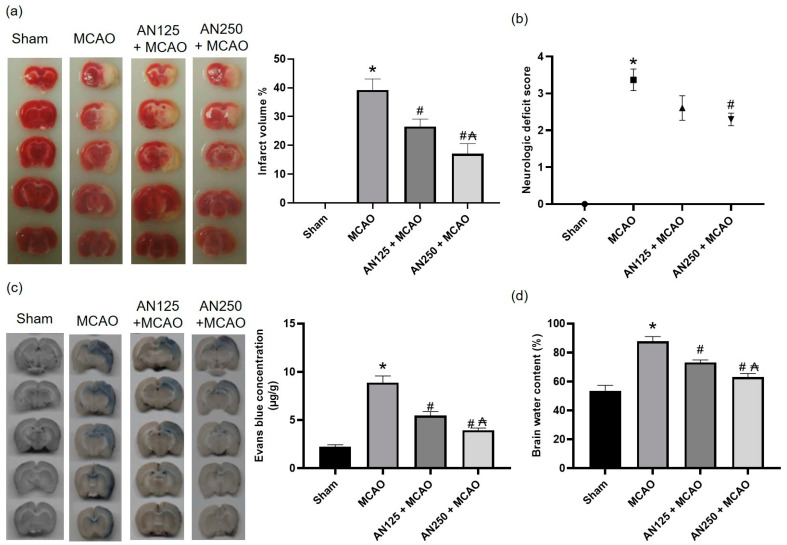
The impact of pretreatment with anethole (AN, 125 and 250 mg/kg, orally) for two weeks in MCAO-induced cerebral ischemia/reperfusion on (**a**) infarct volume, (**b**) neurological deficits, (**c**) Evans blue concentration and (**d**) brain water content percentage. Data are expressed as mean ± SD (*n* = 6). * significantly different from the sham-operated group, # significantly different from the MCAO ischemic group, and ₳ significantly different from the AN125 + MCAO group at *p* < 0.05 using ANOVA followed by Tukey’s post hoc test.

In the current study, first, we examined whether AN protected the brain against ischemia-induced damage associated with 24 h of reperfusion after MCAO or not. TTC-stained brain sections showed an extensive infarct area in MCAO animals, whereas pretreatment with AN (125 and 250 mg/kg) diminished the infarct volume significantly (Figure 1a). In addition, as revealed in Figure 1b, severe neurological deficits were presented in the animals that experienced MCAO, while pretreatment with AN 250 mg/kg reduced the neurological score.

### 2.2. AN Alleviated Behavioral Changes Caused by MCAO-Induced C I/R

Table 1 explained that MCAO rats presented a significant reduction in final locomotor activity and falling latency time as compared to sham rats. Pretreatment with AN in doses of 125 and 250 mg/kg showed a significant increase in final locomotor activity and prolonged the final falling latency time as compared to MCAO rats. However, no difference was shown between the two doses of AN in final locomotor activity and falling latency time.

### 2.3. AN Improved BBB Integrity and Reduced Brain Edema Caused by MCAO-Induced C I/R

The effects of pretreatment with AN on MCAO-induced BBB disruption and the resulting brain edema were investigated. BBB permeability was evaluated using Evans blue staining as manifested in Figure 1c. Evans blue intensity increased in MCAO animals while pretreatment with AN significantly (*p* < 0.05) lowered Evans blue intensity indicating improved BBB integrity as illustrated in Figure 1c. Additionally, the brain water content in the MCAO group was significantly higher than in the sham group, whereas the brain water content in the animals pretreated with AN (125 and 250 mg/kg) was significantly decreased when compared to MCAO group (Figure 1d).

### 2.4. AN Amended Histopathological Alteration Caused by MCAO-Induced C I/R

**Figure 2:**
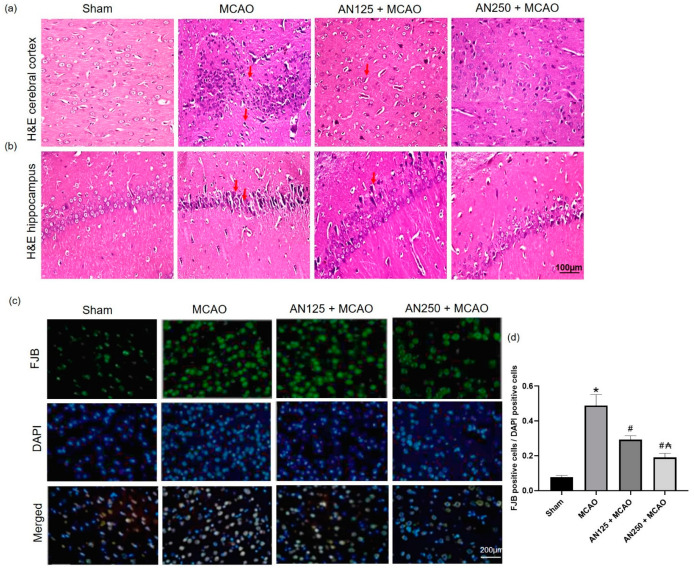
The impact of pretreatment with anethole (AN, 125, 250 mg/kg, orally) for two weeks in MCAO-induced cerebral ischemia/reperfusion on histopathology changes in penumbra area of (**a**) the cortex and (**b**) hippocampus neurons using H&E (×400) and on (**c**) Fluoro-Jade B stained neuronal degeneration and (**d**) FJB positive cells/DAPI positive cells. Data are expressed as mean ± SD (*n* = 6). * significantly different from the sham-operated group, # significantly different from the MCAO ischemic group, and ₳ significantly different from the AN125 + MCAO group.

To further examine the neuroprotective effects of AN, the histopathology changes in the rat cortex and hippocampus neurons were detected by H&E staining. The brain sections attained from sham-operated animals presented no tissue alterations in the cerebral cortex and hippocampus (Figure 2a,b). In the MCAO group, some neurons in the rat cortex and hippocampus were heterogeneously arranged, the cell body contracted triangular or extremely irregular, the cytoplasm was red-stained, the nucleus was pyknotic, and the structure was unclear. However, the hippocampus presented distinct neuronal degeneration of the pyramidal cells with neurophagia. On the other hand, the groups pretreated with AN showed a marked reduction in the number and degree of degeneration, necrosis, and loss of the rat cortex and hippocampus neurons compared with that in the MCAO group (Figure 2a,b).

### 2.5. AN Alleviated Neuronal Degeneration Caused by MCAO-Induced C I/R

Fluoro-Jade B staining is an established marker for neuronal degeneration and indicates damaged neurons susceptible to cell death, as illustrated in Figure 2c. Sham animals exhibited minimal Fluoro-Jade B-positive cells, whereas amplified Fluoro-Jade B-positive cells were detected in the ischemic area of MCAO animals (Figure 2d). On the other hand, AN pretreatment markedly reduced the number of Fluoro-Jade B-positive cells in MCAO-experienced animals (Figure 2d).

### 2.6. AN Reduced MMP-9 and MMP-2 Caused by MCAO-Induced C I/R

**Figure 3:**
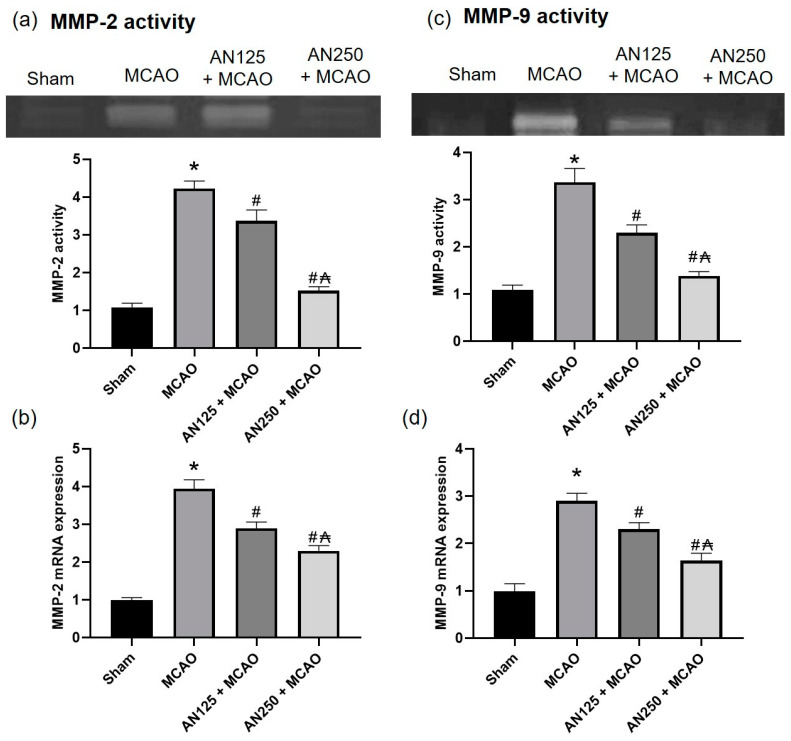
The impact of pretreatment with anethole (AN, 125, 250 mg/kg, orally) for two weeks in MCAO-induced cerebral ischemia/reperfusion in rats on MMP-9 and MMP-2 (**a**,**c**) enzyme activities and (**b**,**d**) gene expression. Data are expressed as mean ± SD (*n* = 6). * significantly different from the sham-operated group, # significantly different from the MCAO ischemic group, and ₳ significantly different from the AN125 + MCAO group at *p* < 0.05 using ANOVA followed by Tukey’s post hoc test.

To investigate whether AN can affect MMP-9 and MMP-2 gene expression and enzyme activities in MCAO-induced injury, PCR and gelatin zymography assays were performed, respectively. MMP-9 and MMP-2 gene expression and enzyme activities were significantly amplified in animals that experienced MCAO. However, pretreatment with AN effectively lowered MMP-9 and MMP-2 gene expression and enzyme activities (*p* < 0.05), signifying that AN may improve BBB integrity via modifying MMP-9 and MMP-2 gene expression and enzyme activities (Figure 3).

### 2.7. AN Reduces Phosphorylation of JNK/P38 Caused by MCAO-Induced C I/R

A potential mechanism for AN’s neuroprotective actions could be through JNK/p38 MAPK pathway. Therefore, phosphorylated JNK and p38 were evaluated via Western blot analysis and immunofluorescence staining.

**Figure 4:**
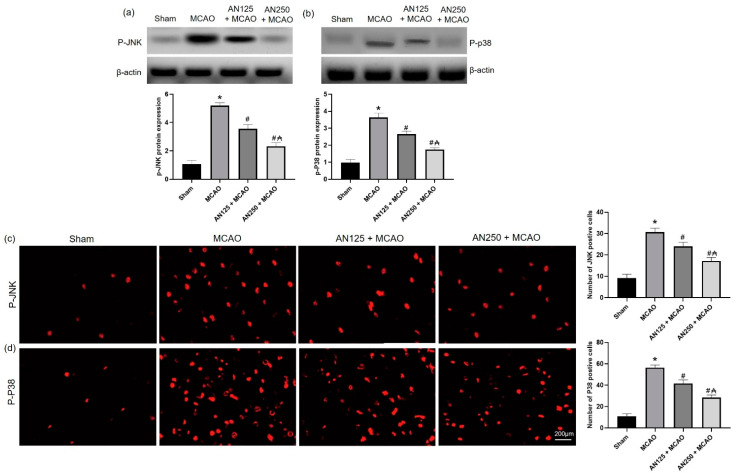
The impact of pretreatment with anethole (AN, 125, 250 mg/kg, orally) for two weeks in MCAO-induced cerebral ischemia/reperfusion in rats on the protein expression of (**a**) p-JNK and (**b**) p-P38 and on the immunofluorescence staining of (**c**) JNK and (**d**) p38 (×400). Data are expressed as mean ± SD (*n* = 6). * significantly different from the sham-operated group, # significantly different from the MCAO ischemic group, and ₳ significantly different from the AN125 + MCAO group at *p* < 0.05 using ANOVA followed by Tukey’s post hoc test.

As illustrated in Figure 4a,b, and compared with the sham-operated group, increased levels of phosphorylated JNK and p38 MAPK were observed in the MCAO group (*p* < 0.05). Compared with the MCAO group, pretreatment with AN significantly decreased the levels of phosphorylated JNK and p38 MAPK. Furthermore, immunofluorescence staining showed an increased number of phosphorylated JNK and p38-positive cells in the MCAO group (Figure 4c,d). However, pretreatment with AN suppressed the number of phosphorylated JNK and p38-positive cells compared to MCAO rats.

### 2.8. AN Alleviated Oxidative Stress Caused by MCAO-Induced C I/R

**Figure 5:**
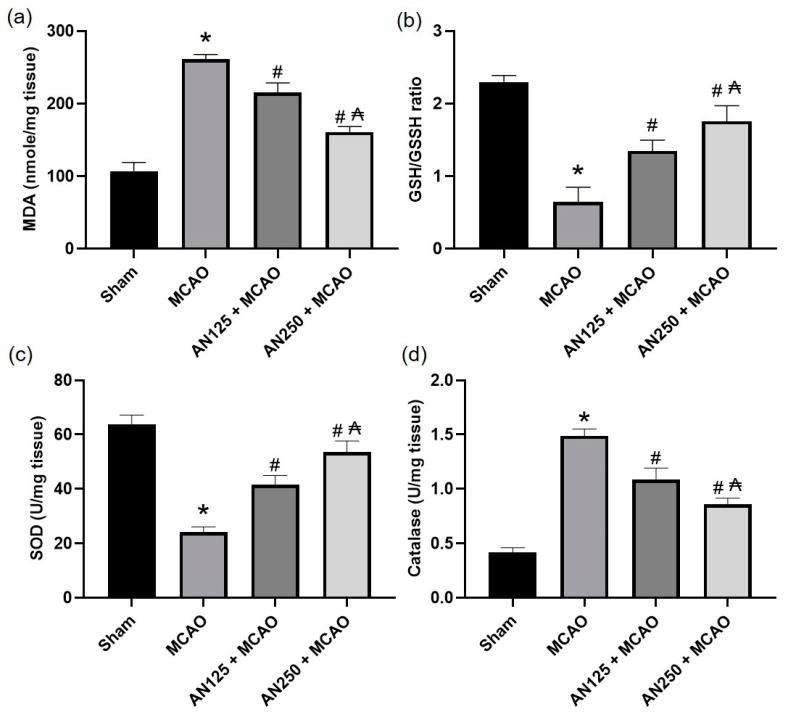
The impact of pretreatment with anethole (AN, 125, 250 mg/kg, orally) for two weeks in MCAO-induced cerebral ischemia/reperfusion in rats on (**a**) MDA (**b**) GSH/GSSG ratio (**c**) SOD and (**d**) catalase contents. Data are expressed as mean ± SD (*n* = 6). * significantly different from the sham-operated group, # significantly different from the MCAO ischemic group, and ₳ significantly different from the AN125 + MCAO group at *p* < 0.05 using ANOVA followed by Tukey’s post hoc test.

Previous studies have indicated that oxidative stress is closely associated with ischemic-perfused cerebral injury [35,36,37]. Thus, the GSH/GSSG ratio, MDA content, and the SOD and catalase content in the ischemic brain homogenate were identified. As displayed in Figure 5, compared with the sham group, the MDA content was significantly amplified, while the SOD and catalase level and GSH/GSSG ratio were diminished considerably in the MCAO-experienced animals’ group (*p* < 0.05). Conversely, pretreatment with AN substantially reversed these modifications, causing a decrease in MDA content as well as an amplified GSH/GSSG ratio and SOD and catalase contents (Figure 5).

### 2.9. AN Alleviated NO Amplification Caused by MCAO-Induced C I/R

NO, as a pre-inflammatory mediator, is derived from NOS and serves an important role in cerebral ischemia and the effects of an ischemic insult [38]. As specified in Table 2, the levels of total NOS (tNOS), induced NOS (iNOS), constitutive NOS (cNOS), and NO in the ischemic hemispheres were significantly elevated in MCAO animals when linked with the sham-operated animals. In addition, AN pretreatment significantly depressed the levels of tNOS, iNOS, cNOS, and NO in the ischemic hemispheres of rats when related to the MCAO group.

### 2.10. AN Reduces NFKB Activation Caused by MCAO-Induced C I/R

**Figure 6:**
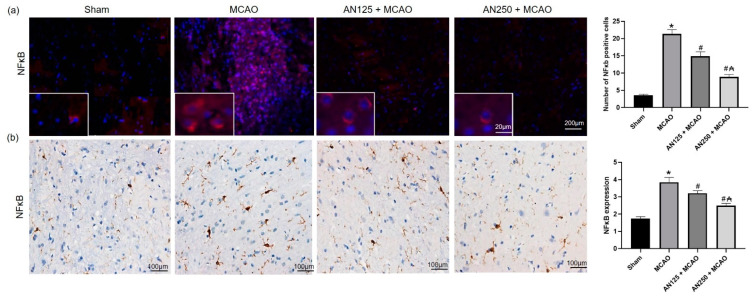
The impact of pretreatment with anethole (AN, 125, 250 mg/kg, orally) for two weeks in MCAO-induced cerebral ischemia/reperfusion in rats on NF-κB/p65 (**a**) immunofluorescence (red cells represent NF-κB/p65) and (**b**) immunohistochemical staining (×400). Data are expressed as mean ± SD (*n* = 6). * significantly different from the sham-operated group, # significantly different from the MCAO ischemic group, and ₳ significantly different from the AN125 + MCAO group at *p* < 0.05 using ANOVA followed by Tukey’s post hoc test.

NF-κB/p65 were assessed via immunohistochemical and immunofluorescence staining. Immunohistochemical and immunofluorescence staining showed that the expression levels of NF-κB were significantly raised in the infarct areas of the brain in the MCAO group (Figure 6). However, pretreatment with AN suppressed the expression of NF-κB compared to rats that did not receive this treatment, as illustrated in Figure 6.

### 2.11. AN Alleviated the Inflammation and Apoptosis Caused by MCAO-Induced C I/R

**Figure 7:**
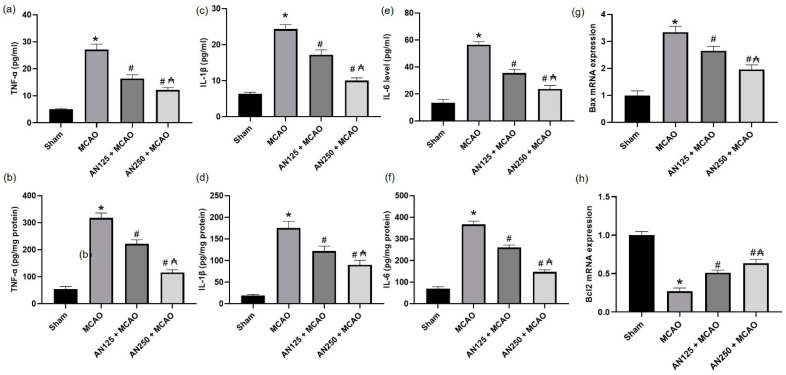
The impact of pretreatment with anethole (AN, 125, 250 mg/kg, orally) for two weeks in MCAO-induced cerebral ischemia/reperfusion in rats on serum and brain tissue homogenate of (**a**,**b**) TNF-α, (**c**,**d**) IL-1β, (**e**,**f**) IL-6 and the gene expression of (**g**) Bax and (**h**) Bcl2, respectively. Data are expressed as mean ± SD (*n* = 6). * significantly different from the sham-operated group, # significantly different from the MCAO ischemic group, and ₳ significantly different from the AN125 + MCAO group at *p* < 0.05 using ANOVA followed by Tukey’s post hoc test.

Ischemic cerebral injury has demonstrated that the inflammatory response plays a critical role; therefore, the current study distinguished the effects of AN on levels of inflammatory cytokines (IL-1β, IL-6, and TNF-α) in serum and brain tissue homogenate. As revealed in Figure 7a–f, the serum and brain tissue homogenate levels of IL-1β, IL-6, and TNF-α were significantly augmented in MCAO animals compared to the sham group (*p* < 0.05). However, pretreatment with AN considerably reversed these alterations in the levels of inflammatory cytokines, causing a decrease in these inflammatory cytokines in the AN-pretreated group (*p* < 0.05) compared with the MCAO group. As for the apoptotic markers, the ischemic animals exhibited a decrease in the gene expression of Bcl2 associated with an increase in the gene expression of Bax, as shown in Figure 7g,h. On the other hand, AN mitigated these alterations.

## 3. Discussion

This study provides unique evidence of the neuroprotective action of AN in MCAO-induced C I/R as manifested in the attenuation of the infarct area, neuronal cell loss, and histopathological damage. According to our knowledge, this is the first research to validate the involvement of behavioral alterations, BBB integrity, oxidative stress, inflammation, and apoptosis through the JNK/p38 pathway in the neuroprotective effect of AN in the C I/R animals model.

In the current study, the MCAO-prompted C I/R animal model resulted in extended infarct volume, severe neurological deficits, reduction in final locomotor activity and falling latency time, and frequent histopathological alterations. These findings agree with preceding studies [39,40] reflecting the development of brain damage with severe neurological and motor dysfunctions in MCAO rats. On the other hand, pretreatment with AN (125 and 250 mg/kg) for two weeks diminished the infarct volume, improved the neurological score, increased final locomotor activity, prolonged the final falling latency time, and enhanced histopathological examination. This finding signifies the neuroprotective ability of AN and is in accordance with earlier studies that displayed AN improved motor performance in rotenone-induced Parkinson’s disease in rats [41]. Fluoro-Jade B-positive cells were amplified in the ischemic area of C I/R animals, suggesting major neuron loss, which is in harmony with earlier reports [42]. However, AN pretreatment markedly reduced the number of Fluoro-Jade B-positive cells in MCAO-experienced animals, demonstrating reduced neuron loss and improved neuronal survival.

Matrix metalloproteinases (MMPs) are elaborated in the pathophysiology of several CNS diseases that share shared pathogeneses, such as the disruption of the blood–brain barrier (BBB), neuroinflammation, and oxidative stress. In early ischemic injury, MMPs participate in BBB interruption by digesting the basal lamina of capillaries and extracellular matrix, leading to vasogenic edema [43]. In the current experiment, C I/R-induced by MCAO amplified Evans’s blue intensity and brain water content, indicating compromised BBB integrity. At the same time, AN effectively lowered Evans blue dye intensity and brain water content, demonstrating improved BBB integrity. We proposed MMPs as one of the elements responsible for the AN enhancement of BBB integrity. Therefore, the gene expressions and enzyme activities of MMP-9 and MMP-2 were evaluated. The results showed that the gene expressions and enzyme activities of MMP-9 and MMP-2 were significantly amplified in animals that experienced C I/R. Preceding studies revealed that MMP-9,2 expressions were significantly amplified in ischemic brains, indicting that MMP augmented the brain injury as well as the BBB breakdown [23,27]. On the other hand, AN lowered MMP-9 and MMP-2 gene expression and enzyme activities, indicating that AN may improve BBB integrity via modifying MMP-9 and MMP-2 gene expression and enzyme activities. Previously, AN inhibited the invasion of DU145 prostate cancer cells and down-regulated the activities of matrix-metalloproteinase (MMPs) [12,44].

The MAPK signaling pathway is involved in inflammatory processes occurring during C I/R injury as it is one of the main signaling mechanisms that regulates neuroinflammation [31,45]. The activated MAPK causes the overproduction of pro-inflammatory factors [46]. In this study, the phosphorylation of JNK and p38 was clearly amplified in MCAO-induced C I/R, indicating that the p-JNK and p-P38 signaling pathways were triggered. Similarly, earlier reports demonstrated the activated JNK and p38 in C I/R [45]. However, AN inhibited the phosphorylation of p-JNK and p-P38, which contributed to lowering the production as well as the release of pro-inflammatory factors. Similarly, AN protects against hepatic I/R injury via suppressing JNK and p38 phosphorylation [47].

Another critical pro-inflammatory enzyme that plays a vital role in BBB disruption is the iNOS, which generates MMP-9 [48]. During cerebral ischemia, iNOS expression elevates, leading to excessive NO production, which results in irreversible cell injury by deterring the mitochondrial respiratory chain and making peroxynitrite with superoxide anions [49]. In addition, several studies showed that ischemia-induced neurotoxicity might be alleviated by iNOS inhibitors [50]. tNOS, iNOS, cNOS, and NO levels in the ischemic hemispheres were elevated in animals suffering from C I/R. AN pretreatment depressed the levels of tNOS, iNOS, cNOS, and NO in the ischemic hemispheres. Several studies showed that AN reduced NO in several non-neurological models such as adjuvant-induced arthritis [51], nonimmune acute inflammation models [8], and in lipopolysaccharide-stimulated RAW 264.7 [52].

Ischemic stroke is associated with oxidative stress due to the excessive production of reactive oxygen species (ROS), which destroy lipids and proteins, resulting in DNA damage in ischemic brain tissue [53,54]. In harmony with prior studies [55,56], the current study outcomes exposed that MCAO-experienced animals exhibited amplified brain MDA content and subsequent reduction in brain content of SOD, GSH/GSSH, and CAT. This antioxidant depletion indicates that oxidative stress intensification mediated neurological dysfunction in C I/R. AN-pretreated animals considerably lessened MDA and amplified SOD, GSH/GSSH, and CAT brain contents, demonstrating the antioxidant activity of AN, which may mediate its neuroprotective effect in C I/R. Consistent with these findings, AN revealed antioxidant effects in neurological disorder such as in rotenone-induced Parkinson disease in rats [41] and in non- neurological disorder such as acetic acid-induced colitis [57] and renal ischemia/reperfusion [58] among other disorders.

Inflammation participates in developing C I/R-induced brain injury, with the associated inflammatory cells and mediators [59]. Furthermore, pieces of evidence reported the associated role of the NF-κB signaling pathway in the ischemic stroke [48,59]. NF-κB regulates the transcription of downstream target genes, including TNF-α, IL-1β, IL-6, and iNOS, which stimulates the manifestation and release of pro-inflammatory factors, thus contributing to neuronal death and exacerbating C I/R injury [48,59]. Consequently, mitigating the NF-κB signaling pathway may alleviate brain damage associated with C I/R. The results of the present study displayed that IL-1β, IL-6, and TNF-α were upregulated in both serum and brain tissue in C I/R-suffered animals. Earlier studies consistently confirmed the imperative role of inflammatory cells and mediators Field [38,60] that were elevated in serum and brain [60]. Pretreatment with AN considerably reversed these alterations in levels of inflammatory cytokines, causing a decrease in the serum and brain tissue homogenate inflammatory cytokines. Several pieces of evidence suggest that AN possesses a robust anti-inflammatory activity via multiple mechanisms. For instance, AN controlled inflammation in adjuvant-induced arthritis in rats [51], acute inflammation induced by carrageenan, and persistent inflammation caused by complete Freund’s adjuvant [2]. Concerning the apoptotic status, the ischemic animals exhibited a decrease in the gene expression of Bcl2 associated with an increase in the gene expression of Bax, revealing activated apoptotic pathways. On the other hand, AN mitigated these alterations, as shown by the decrease in Bax and the increase in the Bcl2 gene expressions.

## 4. Materials and Methods

### 4.1. Middle Cerebral Artery Occlusion (MCAO)-Induced C I/R

Rats were anesthetized with an IP injection of 1% pentobarbital sodium solution (0.45 mL/kg) and were maintained with 1.5% isoflurane and 80% oxygen using an evaporator. The right common carotid artery (CCA), the external carotid artery (ECA), and the internal carotid artery (ICA) were exposed and dissected away from the adjacent nerves and tissues. A filament (18 mm in length) was introduced through a small incision (5 mm) in the CCA and extended to the ICA to cause the Middle cerebral artery (MCA)occlusion, according to Longa, Weinstein [61]. The filament was then withdrawn gently after 120 min of occlusion, allowing blood reperfusion into the brain for 24 h. The same steps were performed in the sham-operation animals, except that the filament was not inserted. Rats were recovered in their cages with unrestricted access to tap water and food. During the MCAO surgery, the animals’ body temperature was conserved at 37 ± 0.5 °C using a thermostatically controlled surgery tray. After the surgery, the animals were kept on the surgery tray till woke up and then were put back in cages with free access to tap water and food.

### 4.2. Animals Attaining and Ethical Code Approval

The Institutional Animal Care and Use Committee of King Faisal University (KFU-REC-2022-OCT-ETHICS213) permitted the experimental protocol. All the experiments were performed consistently with the relevant procedures and regulations of the Ethical Conduct for the Use of Animals in Research at King Faisal University.

### 4.3. Experimental Design

**Figure 8:**
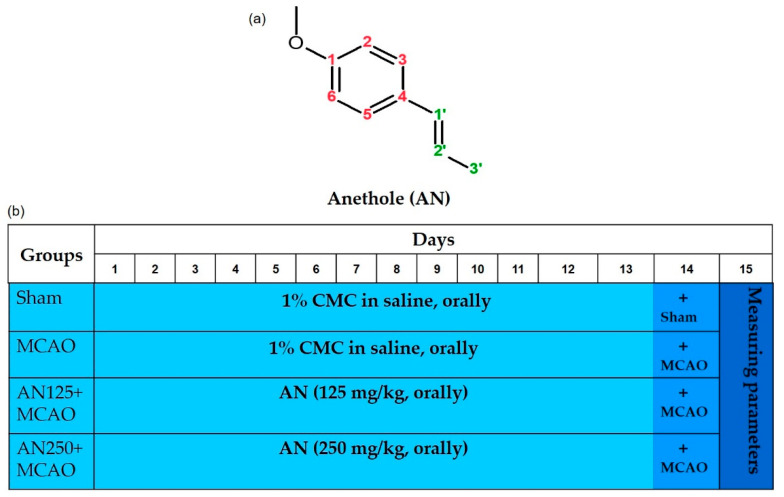
(**a**) Chemical structure of anethole (AN) and (**b**) Experimental design showing the different groups and the respective drug administered.

Sprague–Dawley (SD) male rats (age: 6–8 weeks; weight: 210 ± 30 g) were randomly assigned into four groups: sham, MCAO, AN125 + MCAO, in which animals were pretreated with anethole (AN) 125 mg/kg orally for two weeks before the induction of MCAO. The MCAO operation was performed on the 14th day of the drug administration after 3 h of the drug dose. In the fourth group (AN250 + MCAO), the animals were pretreated with AN 250 mg/kg orally for two weeks before the induction of MCAO. AN (The chemical structure disclosed in Figure 8a) was purchased from Merck & Co., Inc. (Rahway, NJ, USA, product number W208620). AN was originally dissolved in 1% carboxymethylcellulose (CMC) in saline and diluted working solutions (by saline only) were given orally to the animals via gastric lavage. The final volume was 0.5 mL for each oral administration. The whole experimental design is summarized in Figure 8b. The oral doses of AN (125 and 250 mg/kg) were determined depending on previous studies and preliminary experiments performed in our lab [41,62]. Six rats were used for each of the assessments, which required different processing of the brain tissue. For instance, six rats were used for infarct size measurement by TTC staining; 6 rats for BBB permeability assessment by Evans blue dye; 6 rats for evaluating brain water content and six rats for neuronal degeneration assessment and for histopathological, immunohistochemical and immunofluorescence staining. Additionally, 6 rats were used to prepare ischemic brain hemispheres homogenate for RT-PCR, gelatin zymography assay, Western blot assay and biochemical parameters analysis.

### 4.4. Behavioral Tests

#### 4.4.1. Neurologic Deficit Assessment

After 24 h of brain reperfusion, the neurologic deficit was evaluated and executed, as mentioned before [45,63]. The scoring criteria were as follow; Grade 0—no neurological deficit; Grade 1—failure to extend left forepaw fully; Grade 2—constant circling to the left; Grade 3—falling to the left; Grade 4—no spontaneous walking with depressed level of consciousness [64].

#### 4.4.2. Assessment of Spontaneous Locomotor Movement

Rats’ locomotor activity was measured using a grid floor activity cage to detect the rat’s movements. Movements by the rat that interrupted infrared beams were automatically detected, and the beam-interruption information was processed by the activity cage software to provide counts of horizontal movements. Rats were acclimated to the test room for 1 h. Then, rats were placed individually into the activity cage for a 5-min session, and the basal activity counts were recorded. At the end of the session, the rats were removed and returned to their home cage. The area was wiped out with a 70% (*v*/*v*) alcohol solution in distilled water between sessions to prevent olfactory cues. Twenty-four hours after the last administration of the test drugs, each rat was then re-exposed to the activity apparatus for a 5-min test session, and the final activity counts were recorded [65].

#### 4.4.3. Assessment of Motor Coordination

Rats’ motor coordination was assessed using an accelerating rotarod as described before [66]. Animals were trained for three sessions on three consecutive days on the rotarod device at a fixed speed (4 rpm; 4 rotations per minute). On the fourth day, the rats were placed on the testing rod, and the speed of the rotarod started at 4 rpm and then augmented steadily to reach 40 rpm over 300 s. The basal falling time for each rat was recorded using a cut-off limit of 300 s. Twenty-four hours after the last administration of the test drugs, each rat was then re-placed on an accelerating rotarod for 300 s test session, and the final falling time was recorded [55].

### 4.5. Euthanasia, Blood and Brain Tissue Samplings

After 24 h of the last treatment, all animals were euthanized under decapitation and blood was collected, and their brains were removed. Blood was centrifuged to obtain serum, which was used for the biochemical analysis of the antioxidant and inflammatory parameters. Some brain samples were rapidly isolated and frozen, whereas others were kept on 10% neutral-buffered formalin for further investigations. Other ischemic brain hemispheres were randomized into two parts: one for extracting total RNA according to the Trizol kit instructions and another for Western blot and gelatin zymography.

### 4.6. Infarct Volume Assessment

The frozen brains were cut into sections and then incubated in 2% 2,3,5-triphenyltetrazolium chloride (TTC) at 37 °C for 15 min, displaced into 4% paraformaldehyde for overnight [40,45]. Brain infarctions areas were recognized as whitish unstained areas. The infarct volume was scrutinized quantitatively using Image J software using the following formula. Infract volume (%) = (cerebral infarction area/whole brain area) × 100%, as mentioned before in [45].

### 4.7. Brain Water Content Assessment

Brain edema was detected using the wet/dry method as previously described [45]. Briefly, the right brain hemisphere was immediately removed and placed on saline-soaked filter paper to prevent dryness. Pia mater and blood were carefully removed and weighed to obtain the wet weight. Then, the brain tissue was subsequently placed in an oven at 105 °C for 24 h, followed by re-weighing to obtain the dry weight. Brain water content = [(wet weight − dry weight)/wet weight] × 100%.

### 4.8. Blood–Brain Barrier Integrity Assessment

BBB integrity was performed using Evans blue dye, which is one of the vascular permeability markers [67]. Evans blue (EB) dye (100 mg/kg, product number E2129, Merck & Co., Inc., Rahway, NJ, USA) was injected into the femoral vein two hours after the onset of reperfusion. At the end of the reperfusion, rats were perfused with saline through the left ventricle till colorless perfusion fluid was achieved from the right atrium. The brain was removed from the skull, dissected, weighed, and soaked in a 50% trichloroacetic acid solution. After centrifugation (13,600× *g*, 20 min), the supernatant was diluted in anhydrous ethanol three times. The absorbance value was measured by a fluorescence spectrophotometer (excitation wavelength 620 nm, emission wavelength 680 nm). The EB content was calculated from a standard EB curve to measure the change in BBB permeability [45]. The content of EB in brain tissue extract was quantified by microgram per gram of brain tissue.

### 4.9. Determination Using Ischemic Brain Sections

#### 4.9.1. Neuronal Degeneration Assessment

Fluoro-Jade B staining was executed to inspect the neuronal degeneration following MCAO-induced cerebral ischemia-reperfusion, as mentioned in [68,69]. The brain sections were deparaffinized with xylene and rehydrated with ethyl alcohol. Subsequently, the brain sections were incubated with 1% NaOH in 80% ethanol, 70% ethanol, and then distilled water. Sections were reacted with a 0.06% potassium permanganate solution for 10 min and stained with a 0.1% acetic acid solution containing 0.01% Fluoro-Jade B for 30 min. After staining, sections were rinsed with distilled water and dried. Dried brain sections were incubated in 4′,6-diamidine-2-phenylindole (DAPI, product number 32670 Merck) and mounted with dibutyl phthalate polystyrene xylene (DPX) mounting media. Section images were observed and captured with a fluorescent microscope (Leica DM500, Leica, München, Germany). At least ten different fields were photographed from each section, and the images were analyzed by Image J software. For each animal, the total number of cells was averaged across fields of view for cortex, striatum, or both (cortex + striatum). These averages (avg # cells/field of view) were used for statistical analysis. The proportion of Fluoro-Jade B-positive cells was measured by the ratio of Fluoro-Jade B-positive cells to DAPI positive cells.

#### 4.9.2. Histopathological Staining

After 24 h of the MCAO-induced cerebral ischemia/reperfusion, the animals (*n* = 6) were anaesthetized with 10% chloral hydrate (350 mg/kg) and perfused with ice-cold saline and 4% paraformaldehyde. Brains were removed and fixed in 4% paraformaldehyde at four °C for 24h, dehydrated in graded ethanol and xylene, and then embedded in paraffin. The paraffin-embedded brains were serially sectioned into slices of 4 μm with a microtome. Finally, the slices were dewaxed, dehydrated, and stained with hematoxylin and eosin (H&E) for pathological evaluation.

#### 4.9.3. Immunohistochemical Staining

After 24 h of the I/R surgery, immunohistochemical staining was performed [70]. Paraffin sections were prepared as in H&E staining (above). The sections were immersed in 3% hydrogen peroxide (H_2_O_2_) in methanol (21–25 °C, 30 min) and washed with PBS three times. After blocking using 1% normal goat serum blocking buffer for 10 min, the brain sections were incubated with NFκB antibody [71] (1:100, catalogue No. 436700, Thermo Fisher Scientific, Cambridge, UK) overnight at four °C, followed by goat anti-rabbit-horseradish peroxidase (HRP) conjugated IgG antibody (diluted 1:500, Santa Cruz Biotechnology, Santa Cruz, CA, USA) for 30 min. After color development with 3,3′-Diaminobenzidine (DAB) (5 min), the brain sections were counterstained with 1% hematoxylin for (2 min, 21–25 °C), and mounted with neutral gum. The sections were observed and photographed under a ×400 light microscope.

#### 4.9.4. Immunofluorescence Analyses

Paraffin-embedded coronal brain sections were subjected to deparaffinization and rehydration and then underwent a microwave oven antigen retrieval (microwave method). The brain sections were incubated overnight at four °C with the primary antibody: NF-κB (1:1000, catalogue No. ab16502), or P-JNK (1:1000, catalogue No. ab47337), or P-p38 (1:1000, catalogue No. ab4822). JNK and p38 antibodies were obtained from Abcam Biotechnology, Cambridge, MA, USA). The slides were rinsed with cold PBS to remove the unbound antibodies. Sections were then incubated with IgG secondary antibody (1:2000 dilutions, goat anti-rabbit, Abcam Biotechnology) for one h at room temperature followed by 4′,6-diamidino-2-phenylindole (DAPI) for 5 min at room temperature. Finally, the sections were mounted with mounting media, cover-slipped, and air-dried. Cells stained for NFκB in the core ischemic wound of the cerebral tissues were randomly analyzed in 10 sections of each brain under high magnification (20× or 40×). The results were presented as mean ± SD.

### 4.10. Ischemic Brain Hemispheres

#### 4.10.1. RT-PCR Detection

The ischemic brain hemispheres were used for extracting total RNA according to the Trizol kit instructions. The MMLV-RT kit was used to reverse transcribe total RNA into cDNA. The MMP-9 primer (5′-AAATGTGGGTGTACACAGGC-3′, 3′-TTCACCCGGTTGTGGAAACT-5′), MMP-2 (5′-TGCCATCCCTGATAACCTG-3′, 3′-CAGCCAGTCCGATTTGATG-5′), Bcl-2 (5′-CCGGGAGATCGTGATGAAGT-3′, 3′-ATCCCAGCCTCCGTTAT CCT-5′), Bax (5′-GTGGTTGCCCTCTTCTACTTTG-3′, 3′-CACAAAGATGGTCACTGTC TGC-5′) and the internal reference β-actin primer (5′-ATC CTG CGT CTG GAC CTGG-3′, 5′-TTG GCA TAG AGG TCT TTA CGG AT-3′) were amplified by PCR. In a 25 μL reaction volume, PCR was performed as follows: initial denaturation for 4 min at 94 °C, followed by denaturation for 30 s at 94 °C, annealing for 30 s at 62 °C, and extension for 2 min at 72 °C for 35 cycles, and final extension for 10 min at 72 °C. qPCR was applied using an SYBR ExScript RT-PCR kit, and quantification examinations were accomplished via an Opticon-2 Real-time PCR reactor (MJ Research, Capital Court, Reno, NV, USA). qPCR results were obtained using Step PE Applied Biosystems (Waltham, MA, USA) software. Relative gene expression data were calculated using the (2^−∆∆Cq2^) method and presented as a fold change [72]. Target gene expressions were assessed and related to the reference gene, and the results were presented in the figures as relative expressions.

#### 4.10.2. Gelatin Zymography Assay

The brain hemisphere samples obtained from different groups were homogenized in lysis buffer, including protease inhibitors at 50 mg/mL, and then centrifuged at 12,000 rpm for 15 min at four °C. The activity of MMP-9 and MMP-2 was assessed as mentioned earlier [45] using a gelatin zymography kit obtained from Abcam Inc. (ab234057, Cambridge, UK) following the manufacturer’s protocol.

#### 4.10.3. Western Blot Analysis

First, for the determination of protein levels, frozen brain tissues (cortex) were homogenized with total protein extraction lysis buffer (Beijing Solarbio Science and Technology Co., Ltd., Beijing, China). The protein concentration was determined using a Bio-Rad protein assay (Bio-Rad Laboratories Inc., Hercules, CA, USA), as mentioned before [73]. Western blotting was performed as previously described [45]. Briefly, cortical tissues were homogenized in a buffer and centrifuged at 15,000× *g*. The supernatant was collected, immediately lysed in sodium dodecyl sulfate (SDS)-sample buffer (50 mM Tris–HCl, pH 6.8, 2% SDS, and 10% glycerol), boiled, and reduced with β-mercaptoethanol. Protein samples were loaded (10 or 20 μg/lane), separated in 10% SDS polyacrylamide gels, and then transferred to nitrocellulose membranes (15 V, 50 min; Bio-Rad Laboratories Inc., Hercules, CA, USA). Membranes were blocked with 5% bovine serum albumin (BSA) in Tris-buffered saline (TBS) containing 0.1% Tween-20 (TBS-T) at room temperature for one h. The membranes were incubated with the primary antibodies, diluted in blocking buffer, at four °C overnight: p-JNK monoclonal antibody (1:2000; Cat. No: 4376, Cell Signaling Technology, Danvers, MA, USA), p-p38 monoclonal antibody (1:3000; Cat. No: 4511, Cell Signaling Technology, Danvers, Massachusetts, USA). After ten washes with TBS-T for 3 min each, membranes were incubated for one h at room temperature with the secondary antibody, horseradish peroxidase (HRP)-conjugated anti-rabbit IgG (1:10,000; cat. no. SA000012; Proteintech Group, Inc., Manchester, UK). After ten washes with TBS-T for 3 min each, immunoreactive bands were visualized using a Light-Capture with ECL™ Western blotting analysis system. β-actin was used as an internal control. The signal intensity of immunoreactive bands was analyzed using Image Lab software (Bio-Rad, Hercules, CA, USA).

#### 4.10.4. Determination of NO Content and the Activities of the Total NOS (TNOS), Induced NOS (iNOS), and Constitutive NOS (cNOS) in Brain Homogenate

NO assay kit (ab65328, Abcam Co., Waltham, MA, USA) analyzed the content of NO in brain tissue homogenate, following the manufacturer’s instructions. The activities of TNOS (MBS723386), iNOS (MBS263618), and constitutive/endothelial nitric oxide synthase (MBS160509) were measured using kits obtained from MyBioSource consistent with the manufacturer’s instructions.

#### 4.10.5. Determination of Oxidative Stress

Malondialdehyde (MDA; ab238537) and GSH/GSSG ratio (ab138881) kits were acquired from Abcam Inc. (Cambridge, UK). Superoxide dismutase (SOD; MBS036924) and catalase (CAT; 726781) ELISA kits were obtained from My BioSource (San Diego, CA, USA). All the procedures were executed using brain homogenates samples and in agreement with the manufacturer’s directions.

#### 4.10.6. Measurement of Inflammatory Mediators

Inflammatory mediators comprising IL-1β (ab100768), IL-6 (ab100772), and TNF-α (ab46070) ELISA kits were measured in ischemic brain hemisphere homogenates as well as in serum. Kits were attained from Abcam Co., Eugene, OR, USA.

### 4.11. Statistical Analysis

Data are presented as mean ± SD. For multiple comparisons, one-way ANOVA followed by Tukey–Kramer as a post hoc test was performed. For non-parametric data, the Kruskal–Wallis test was used. The 0.05 level of probability was used as the significance level. All statistical analyses were performed using Graph Pad software (version 5, San Diego, CA, USA).

## 5. Conclusions

In conclusion, this study revealed the neuroprotective ability of AN in MCAO-induced C I/R. AN attenuated the infarct area, neuronal cell loss, behavioral alterations, and histopathological changes. AN boosted BBB integrity via modulating MMPs and diminished oxidative stress, inflammation, and apoptosis through JNK/p38 pathway. Our findings may aid in the discovery of new therapeutic options based on AN in the prevention/mitigation of ischemic stroke-induced brain injury. This research is considered an addition to the scientific pool of AN, the well-known phenylpropene components of many plants’ essential oils.

## Figures and Tables

**Table 1:** The impact of pretreatment with anethole (AN, 125, 250 mg/kg, orally) for two weeks in MCAO-induced cerebral ischemia/reperfusion in rats on spontaneous locomotor activity using the activity cage test and on motor coordination using the rotarod test.

Groups	Locomotor Activity (Counts/5 min)		Falling Latency Time (min)	
	Basal	Final	Basal	Final
Sham	150.00 ± 20.47	80.00 ± 4.57	5.01 ± 0.41	4.74 ± 0.35
MCAO	142.75 ± 12.93	27.92 ± 1.63 *	4.95 ± 0.54	2.19 ± 0.36 *
AN125 + MCAO	146.75 ± 15.42	56.38 ± 8.32 #	4.76 ± 0.36	3.57 ± 0.52 #
AN250 + MCAO	125.60 ± 16.35	59.25 ± 7.40 #	4.85 ± 0.44	3.86 ± 0.47 #

Data are expressed as mean ± SD (*n* = 6). * significantly different from the sham-operated group, # significantly different from the MCAO ischemic group.

**Table 2:** The impact of pretreatment with anethole (AN, 125, 250 mg/kg, orally) for two weeks in MCAO-induced cerebral ischemia/reperfusion in rats on the levels of total-, induced- and constitutive nitric oxide synthase and the content of nitric oxide in brain tissues of rats with middle cerebral artery occlusion.

Group	tNOS (U/mg Tissue Protein)	iNOS (U/mg Tissue Protein)	cNOS (U/mg Tissue Protein)	NO (µmol/g Tissue Protein)
Sham	2.13 ± 0.53	0.39 ± 0.13	0.92 ± 0.12	6.34 ± 2.21
Model	3.47 ± 0.42 *	0.96 ± 0.21 *	2.45 ± 0.22 *	19.26 ± 2.6 *
AN 125 + MCAO	2.86 ± 0.34 #	0.61 ± 0.17 #	1.59 ± 0.28 #	12.67 ± 2.30 #
AN 250 + MCAO	2.47 ± 0.23 #₳	0.46 ± 0.14 #₳	1.08 ± 0.13 #₳	9.57 ± 3.2 #₳

Data are expressed as mean ± SD (*n* = 6). * significantly different from the sham-operated group, # significantly different from the MCAO ischemic group, and ₳ significantly different from the AN125 + MCAO group at *p* < 0.05 using ANOVA followed by Tukey’s post hoc test. NOS: nitric oxide synthase, tNOS: total NOS, iNOS: induced NOS, cNOS: constitutive NOS, NO: nitric oxide.

## Data Availability

The data presented in this study are available in the article.
